# Normalisation against Circadian and Age-Related Disturbances Enables Robust Detection of Gene Expression Changes in Liver of Aged Mice

**DOI:** 10.1371/journal.pone.0169615

**Published:** 2017-01-09

**Authors:** Sara S. Fonseca Costa, Daniel Wegmann, Jürgen A. Ripperger

**Affiliations:** 1 Department of Biology, University of Fribourg, Fribourg, Switzerland; 2 Swiss Institute of Bioinformatics, University of Lausanne, Lausanne, Switzerland; Montana State University Bozeman, UNITED STATES

## Abstract

The expression of some genes is affected by age. To detect such age-related changes, their expression levels are related to constant marker genes. However, transcriptional noise increasing with advancing age renders difficult the identification of real age-related changes because it may affect the marker genes as well. Here, we report a selection procedure for genes appropriate to normalise the mouse liver transcriptome under various conditions including age. These genes were chosen from an initial set of 16 candidate genes defined based on a RNA-sequencing experiment and published literature. A subset of genes was selected based on rigorous statistical assessment of their variability using both RNA-sequencing and Nanostring hybridization experiments. The robustness of these marker genes was then verified by the analysis of 130 publicly available data sets using the mouse liver transcriptome. Altogether, a set of three genes, *Atp5h*, *Gsk3β*, and *Sirt2* fulfilled our strict selection criteria in all assessments, while four more genes, *Nono*, *Tprkb*, *Tspo*, and *Ttr* passed all but one assessment and were included into the final set of marker genes to enhance robustness of normalisation against outliers. Using the geometric mean of expression of the genes to normalise Nanostring hybridization experiments we reliably identified age-related increases in the expression of *Casein kinase 1δ* and *1ϵ*, and *Sfpq*, while the expression of the glucose transporter *Glut2* decreased. The age-related changes were verified by real-time PCR and Western blot analysis. As conclusion, proper normalisation enhances the robustness of quantitative methods addressing age-related changes of a transcriptome.

## Introduction

Ageing is the ultimate threat to the survival of an organism and is developing into a health problem for the society because many more people than before reach an age closer to the maximal life expectancy [1]. Transcriptional programmes mediate some changes during ageing optimizing the metabolism and physiology for the needs of an older organism. However, some of these programmes also provoke the progressive accumulation of damage, which finally exceeds the decreasing repair capacity of the body [2]. Interestingly, some processes, for example the process generating circadian rhythms are speculated to play a part during the ageing process. Understanding the potential interactions between the circadian clock and the ageing process could, therefore, offer new strategies to delay the adverse effects of ageing [3] [4].

Circadian rhythms are generated by interlocked transcriptional and post-translational feedback loops driving 24 h rhythmic gene expression [5]. In mammals, complexes of PERIOD (PER) and CRYPTOCHROME (CRY) proteins generate overt daily rhythms by counterbalancing the transcription factors Brain and Muscle ARNT-Like 1 (BMAL1) and Circadian Locomotor Output Cycles Kaput (CLOCK) [6]. On the other hand, *Bmal1* and *Clock* rhythmic expression is mediated by the activating and repressing activity of nuclear receptors of the RAR-related Orphan Receptors (ROR) and Peroxisome Proliferator-Activated Receptors (PPAR), and REV-ERB families, respectively [7] [8] [9]. The process is further fine-tuned by post-translational regulation such as phosphorylation of PER by the Casein kinase (Csnk) 1*δ* and 1*ϵ* [10] [11] to affect stability and cellular location of the proteins adjusting the period length. To achieve a functional output, the circadian oscillator drives rhythmic expression of multiple families of transcriptional regulators such as those from the Drosophila Behaviour/ Human Splicing (DBHS) family [12].

The mammalian timing system governs many metabolic, physiological and behavioural aspects of the daily life [13]. Consequently, external or internal factors modulating this coupled system might have widespread consequences. Previously, it was shown that aged organisms display impaired circadian rhythms in regions of the brain [14] and in vitro in fibroblasts [15] due to reduced neuronal synchronization and yet unknown blood-borne factors, respectively. Despite this, no gross aberration of the underlying circadian oscillator with advancing age has been reported to date. This discrepancy may likely be due to the difficulty in detecting such changes because of the noisy nature of age-related gene expression [16] and the lack of an established protocol to normalise expression data with age-resistant markers. Historically, housekeeping genes have been used for this purpose including metabolic enzymes such as *Glyceraldehyde-3-phosphate dehydrogenase* (*Gapdh*) and the cytoskeleton component *β*-*actin* [17]. However, in some tissues, these genes are not as constantly expressed with advancing age as previously thought [18]. Also, an intrinsic problem for normalisation is the noise of gene expression that can only be overcome by vigorous statistical testing [19].

Here we used centered log-ratio analysis [20] to solve the problem of noise in circadian and age-related gene expression. From our analyses, we selected a total of seven genes and used their geometric mean of expression for normalisation. With the obtained increase in robustness, we were able to identify age-related changes within the circadian regulatory network. Hence, part of the circadian oscillator in the liver is affected by progressing age, which may impact the orchestration of metabolism and physiology.

## Materials and Methods

### Animals and ethics statement

Animal care and handling was performed according to the Swiss Law for Animal Experimentation (TschG, SR455) and the declaration of Helsinki as authorized by the Office Vétérinaire Cantonale de Fribourg (*No*.2013_32_*FR*) and approved by the cantonal veterinarian office of the Canton of Fribourg. Male C57BL/6Rj mice were obtained from a special breeding program maintained at Janvier (St. Berthevin, France). The median life expectancy of these mice is about 28 months and their life span about 30 to 32 months [21] [22] [23]. Consequently, mice with an age of 3 months we considered as young, mice of 12 months as middle-aged, and of 24 months as aged, because they showed first signs of senescence. The mice were kept with water and food ad libitum, and a light schedule of 12 h light/12 h dark. Zeitgeber time (ZT) is defined as ZT0 = lights on, and ZT12 = lights off. Animals were sacrificed under 3% isofluorane/oxygen anesthesia by surgically removing their heads.

### Tissue acquisition and RNA extraction

We isolated RNA from livers of mice up to 24 months old. RNA from homogenized liver was isolated using the NucleoSpin RNA kit from Machery and Nagel (Düren, Germany) and quantified with a Nanodrop 1000 spectrophotometer (NanoDrop Products, Wilmington DE) at 260 nm. The integrity of the RNA samples was verified on 1% agarose bleach gels [24].

### RNA-sequencing

For the RNA-sequencing experiment, RNA from four mouse livers taken at ZT2, ZT6, ZT10, ZT14, ZT18 or ZT22 was taken from 3, 9, 15 or 21 month-old mice (a total of 24 mice per age class). After extraction and for each age class, 1 μg of the total RNA from each individual sample was pooled, resulting in four combined samples of 24 μg total RNA. The RNA in these pools was then digested with 5’-phosphate-dependent RNAse to remove uncapped RNA and the remaining mRNA purified. After cleavage of the 5’-cap, the bar-coded sequencing linkers were added, the library amplified and subjected to massive-parallel SOLiD sequencing [25]. Forward sequences (35 bp) for all four samples were produced on one lane using the SOLiD 5,500 xl sequencing platform. The obtained reads were processed with the Genomic workbench (CLC Bio, Aarhus, Denmark) and mapped to the annotated Mus musculus mm9 reference sequence http://hgdownload.cse.ucsc.edu/goldenPath/mm9/bigZips/chromFa.tar.gz using these stringent conditions (similarity of 0.95 and length fraction of 0.95, i.e. allowing roughly one mismatch or size difference). The mappings were subsequently verified using the IGV2.0 integrative Genomics Viewer (IGV, Broad Institute, Cambridge MA). We focused only on genes with annotated first exon and omitted from the analysis un-annotated peaks, peaks in small noncoding RNAs, intronic peaks, and anti-sense transcription start sites. The number of reads per transcription start site was extracted from genes that were present at least 20 times in one of the libraries. We identified a total of 1,444 5’-ends of mRNAs to be used in this study (S1 Table).

### Nanostring hybridization

The Nanostring technology allows for the detection of individual RNA molecules without an amplification bias [26]. Here we used this technology to quantify expression levels of 52 genes using probes designed and synthesized by Nanostring consisting of complementary DNA probes spanning exon-exon boundaries to map specifically to mRNA (S2 Table). Each probe consisted of a pair of a 5’-primer bearing a biotin label and a 3’-primer bearing a bar code in form of a specific fluorescent tag. If both primers hybridize to their corresponding mRNA, then the fluorescent tag can be bound to a streptavidin-coated support to identify its probe-specific label. We conducted Nanostring quantification from liver tissue from animals of eight different age groups: 3, 6, 9, 12, 15, 18, 21 and 24 months. Per age group, we took samples from twelve evenly spaced time points (ZT0 to ZT22 with 2 h intervals), resulting in 24 samples quantified individually. For each sample, 300 ng of RNA were hybridized to a pool of Nanostring oligo pairs at the Genomics Platform of the university of Lausanne, Switzerland. Each hybridization experiment was conducted in the presence of positive controls of different concentrations to properly normalise the counts of mRNA to concentrations in the original sample. Specifically, and following recommendations from [26], we first inferred the proper normalisation constant cs;*i* for each sample s and for each positive control *i*. We then combined these individual measures into a single normalisation constant ci by taking the geometric mean across all individual constants for that sample.

### Analysis of RNA-sequencing experiments from literature

We downloaded RNA-sequencing data of 3 previously published RNA-sequencing experiments [27] [28] [29] with the reference numbers GEO:GSE57809,GEO:GSM723772 and GEO:GSE48109 from the Geo Omnibus database. The raw reads were mapped and aligned to the mouse genome (UCSC version mm9) using Tophat [30], and uniquely mapped sequences from the output files were then used to obtain read counts using HTseq-count [31].

### Analysis of data mining from literature

We used RNA-sequencing and DNA microarray data from the database Genevestigator [32] with a total of 380 studies using mouse with 9,411 samples, 814 conditions and 339 different genotypes. 130 experiments concerning liver gene expression were exported. We performed gene perturbation analysis on our selection of 16 genes using a cumulative distribution function.

### Centered log-ratio analysis

Log-ratio transformations are the method of choice for distance-based analysis of compositional data [20]. Here we conducted centered log-ratio transformations to compare genes in their variability and overall level of expression. Let *x*_*i*_ = *x*(1)_*i*_, …, *x*(*N*)_*i*_ be a vector of expression measurements for *N* genes under condition *i* (e.g., a specific age group). The so called centered log-ratio (*clr*) is then defined by: *clr*(*xi*) = *log*(*x*(1)_*i*_/*g*(*x*_*i*_)), …, *log*(*x*(*N*)_*i*_/*g*(*x_i_*)); where *g*(*x*_*i*_) is the geometric mean of the original data vector *xi*. The *clr* analysis is related to the fold-change analysis frequently applied to expression data. Specifically, the distance between clr transformed expression levels of the same gene corresponds to the logarithm of the fold-change if the original datasets were normalised such that they share the same geometric mean. Calculating the variance in the *clr* expression levels across many conditions is thus a natural extension of the fold-change analysis to more than two comparisons.

### Repeated-measures ANOVA

To perform the analysis of two different ages at all time points we used the R software (http://www.R-project.org/) and specifically the lme function to calculate the repeated-measures ANOVA for the two data sets. Since we cope here with a multiple comparisons situation, a Bonferroni’s correction of the p-value should be performed, i.e. the p-value to indicate significance would be 0.05/36 = 0.0013888.

### Two-way ANOVA

To estimate the influence of interaction, age and time on the data sets obtained from young and very old animals we used the program PRISM5 (GraphPad software, San Diego, CA). A Bonferroni post test was performed to compare the column pairs and significant changes indicated (*: p < 0.05, **: p < 0.01, ***: p < 0.001).

### Quantification of specific genes by real-time PCR

Reverse-strand cDNA was synthesised using Superscript II (Life Technologies, Carlsbad CA) starting from 0.5 *μg* of total RNA and specific genes detected by real-time PCR with specific primer/probe combinations (S3 Table). For normalisation we used the geometric mean of expression of *Atp5h*, *Gsk3β*, and *Sirt2*.

### Preparation of protein extracts

Liver nuclei were prepared by centrifugation through 2M sucrose cushions as described [33] and liver nuclear extracts prepared. About 20 *μg* of nuclear extract was separated on 7% SDS-polyacrylamide gels, transferred to BA83 nitrocellulose membranes and Western blot performed. Antibodies used were anti-RNA polymerase II (ab817, Abcam, Cambridge, UK), anti-Sfpq (kind gift from Steven Brown, Zurich), and anti-Csnk1*δ* (sc-55553, Santa Cruz Biotechnology, Dallas, TX). Specific antibody:antigen complexes were detected with matching HRP-conjugated secondary antibodies and Western Bright Sirius enhanced chemiluminescence (Advansta, Mento Park, CA) using an Azure C500 machine (Azure Biosystems, Dublin, CA) and increasing exposure times of 30 sec each. All images were analysed using ImageJ (http://imagej.nih.gov/ij/index.html).

## Results

### Rationale for the selection of candidate genes

During the analysis of age-related changes in the liver transcriptome, we realized that these changes were not reliably detectable due to transcriptional noise. To overcome this experimental problem, we therefore began by manually curating a set of potential candidate genes for normalisation. We included 9 genes selected from a pilot RNA-sequencing experiment comparing 3 with 15 month-old animals (S1 Table). To ensure that differences between the age classes were not affected by differences in daily rhythms, we pooled RNA extracted from six different time points throughout the day. The genes were chosen based on the following two criteria: i) their expression was sufficiently large to ensure accurate quantification, and ii) they showed a similar fold-change as *Gapdh*, a gene generally used to normalise gene expression in this tissue [17]. The genes selected this way were *Aox3*, *Atp5h*, *Gapdh*, *S100a1*, *Tspo*, *Ttr*, *Tomm7*, *Tprkb* and *Pqbp1*.

We then complemented this list with the two genes *H3f3a* and *H3f3b* encoding for the histone H3 variant H3.3 and the gene *Nr3c1*/*Gr*, since the accumulation of these classes of proteins was previously described to be unaffected by age [34] [35]. Finally, we added four genes we suspected to be stable across age from experience of working with circadian gene expression over many years: *Gsk3β*, *Nono*, *Rorα*, and *Sirt2*. We then set out to assess the properties of these 16 genes more rigorously and systematically by comparing their expression profiles across age with those of other genes using RNA-sequencing and Nanostring hybridization experiments, as well as public data.

### Selection of marker genes by RNA-sequencing experiment

**Fig 1:**
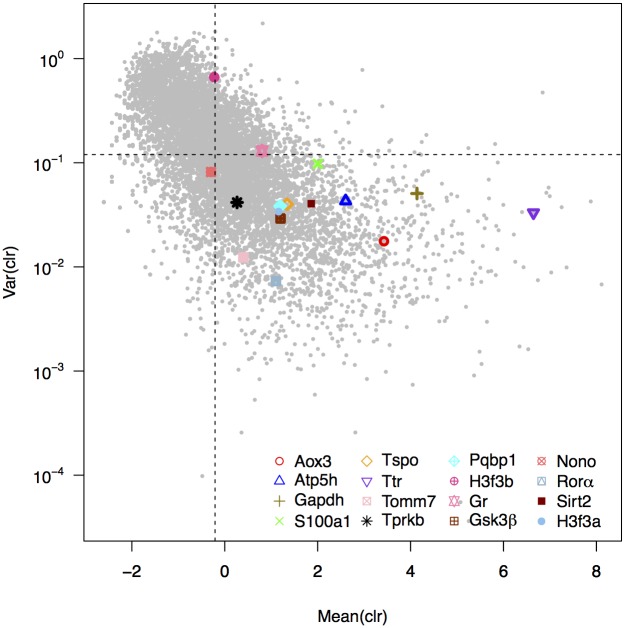
Analysis of the variance of expression of the candidate genes using RNA-sequencing data. For each gene was calculated the variance and mean across four experimental conditions (3, 9, 15 and 21 month-old mice) of the centered log-ratio (*clr*) of their expression (grey dots). The 16 candidate genes are highlighted and the median variance clr and median mean clr across all genes are shown as dashed lines.

To monitor the expression of our candidate genes over age, we extended the RNA-sequencing experiment by comparing gene expression in liver tissue of mice of 3, 9, 15 and 21 months of age. To assess variability in gene expression across age for each gene in the transcriptome, we conducted centered log-ratio analysis, which is used for the analysis of compositional data such as derived from RNA-sequencing and Nanostring hybridization experiments and particularly suitable in the absence of known normalisation markers [20]. This analysis revealed a spectrum of variability as quantified by the variance in centered log-ratios (*clr*) with some genes showing more than 3 orders of magnitude more variability across age classes than others (Fig 1).

Genes also differed greatly in the average expression level, as measured by the mean clr across ages (Fig 1). As confirmed, most of our candidate genes showed little variation and generally large expression levels. Some of our candidate genes, however, showed surprisingly large variation across age classes, in particular the gene *H3f3b*. To further illustrate this, we calculated pair-wise fold-changes between different ages (6 combinations), which also highlighted considerable variation in *H3f3b* (S1 Fig). We considered candidate genes to pass this initial assessment if their average gene expression was above and their variance in expression below the median of these measures of all other genes in the genome. Out of all 16 genes considered, 13 passed these criteria: *Aox3*, *Atp5h*, *Gapdh*, *Gsk3β*, *H3f3a*, *Pqbp1*, *Rorα*, *S100a1*, *Sirt2*, *Tomm7*, *Tprkb*, *Tspo*, and *Ttr*.

### Selection of marker genes by Nanostring hybridization experiment

We next compared the expression profiles of our candidate genes by an independent experiment using Nanostring hybridization. Since Nanostring hybridization experiments can only be conducted on a relatively small, predefined set of genes, we chose to compare all our candidate genes against i) known circadian genes, ii) genes known to modify the circadian oscillator, as well as iii) genes the expression of which is directly affected by the circadian oscillator. The Nanostring hybridization experiment, however, allowed us to increase the resolution of our assessment by doubling both the number of age classes as well as time points per day, and to measure 52 individually measured probe sets (S2 Table).

**Fig 2:**
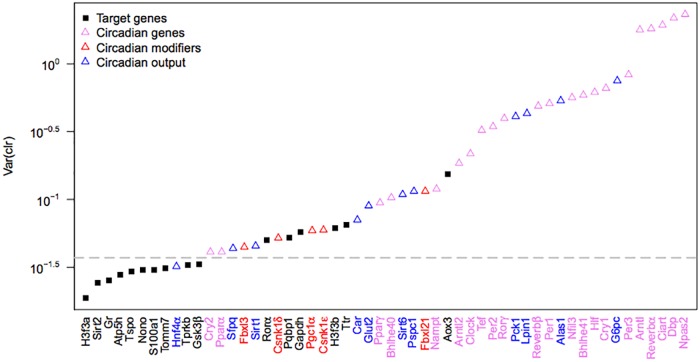
Analysis of the variance of expression of the candidate genes using Nanostring hybridization data. The variance of the *clr* of the expression level for each gene was calculated across all experimental conditions, i.e. eight different ages and twelve time points each in duplicate (N = 192). Genes are sorted from lowest to highest variance and the cut off to pass the assessment is indicated by a dashed line.

To discriminate between genes with nearly constant and variable expression, we again calculated the variance in the *clr* across individual samples. As expected, most of our candidate genes showed variances very close to zero, and much lower variances than all other genes measured along (Fig 2). We considered candidate genes to pass this assessment if their variance of centered log-ratios was lower than that of the least variable circadian gene *Cry2* (Fig 2). This way we identified 11 out of 16 candidate genes to be stable across age classes (Table 1).

**Table 1:** Summary of quality assessments.

Gene	RNA-Seq	Nanostring	GSE57809	GSM723772	GSE48109	Data Mining
*Atp5h*	X	X	X	X	X	X
*Gsk3β*	X	X	X	X	X	X
*Sirt2*	X	X	X	X	X	X
*Nono*	-	X	ND	X	X	X
*Tprkb*	X	X	X	X	X	-
*Tspo*	X	X	X	X	X	-
*Ttr*	X	-	X	X	X	X
*Gr*	-	X	ND	X	X	-
*H3f3a*	X	X	-	-	X	X
*Pqbp1*	X	-	ND	X	X	-
*Rorα*	X	X	ND	X	-	-
*S100a1*	X	X	-	X	X	-
*Aox3*	X	-	X	X	-	-
*H3f3b*	-	-	X	X	X	-
*Tomm7*	X	X	-	X	-	-
*Gapdh*	X	-	-	X	-	-

X: passed; -: failed; ND: not detectable.

The expression of the genes considered stable in this experiment were highly correlated (all between 0.35 and 0.86, S2 Fig), further corroborating that the variance found is largely due to experimental noise that is affecting these genes very similarly, and hence can be controlled for by using these genes for normalisation. In contrast, expression levels were not or only marginally correlated between genes we found to exhibit large variation (S2 Fig).

### Validation with publicly available RNA-sequencing data

**Fig 3:**
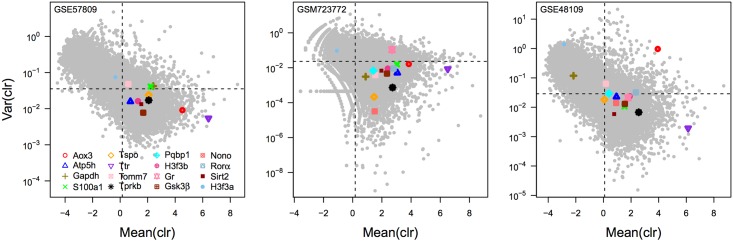
Centered log-ratio analysis of three publicly available RNA-sequencing experiments. We conducted *clr* analysis on three publicly available RNA-sequencing experiments that compared the liver transcriptome of 3 and 21 month-old mice [GEO: GSE57809], between different mouse tissues [GEO:GSM723772], and the liver transcriptome of male and female mice [GEO:GSE48109]. For each study, the variance and mean across all experimental conditions of the clr of the expression is plotted for each gene. The 16 candidate marker genes are highlighted and the median variance clr and median mean clr across all genes are shown as dashed lines.

To further strengthen our results, we assessed the stability of expression of our candidate genes in data from three publicly available RNA-sequencing experiments. These experiments compared gene expression in i) the liver of 3 and 21-month old mice, ii) between many different mouse tissues, or iii) between liver derived from male and female mice. These experiments were chosen because they allowed us to both replicate as well as generalize results from our own data. Just as for our own experiments, we calculated the variance and mean of the clr across replicates for each experiment. In line with our previous results, most of our candidate genes were found to exhibit little variance across replicates but above median expression levels (Fig 3). However, some genes were surprisingly outliers and showed much elevated variation under specific conditions. The expression of the genes *Gapdh*, *H3f3a* and *Aox3*, for instance, were found to vary greatly between males and females. Similarly, the gene *Gr* was found to be very variable across different tissues. Applying the same filters as above, we considered all genes to pass these assessments if their expression levels were above median, but their variance in expression below median when compared to the set of all other genes measured in each study. Only 10 genes passed these requirements in all of the three data sets in which they were measured: *Atp5h*, *Gsk3β*, *Gr*, *H3f3b*, *Nono*, *Pqbp1*, *Sirt2*, *Tprkb*, *Tspo*, and *Ttr*.

### Analysing the stability of the marker genes by meta-analysis

**Fig 4:**
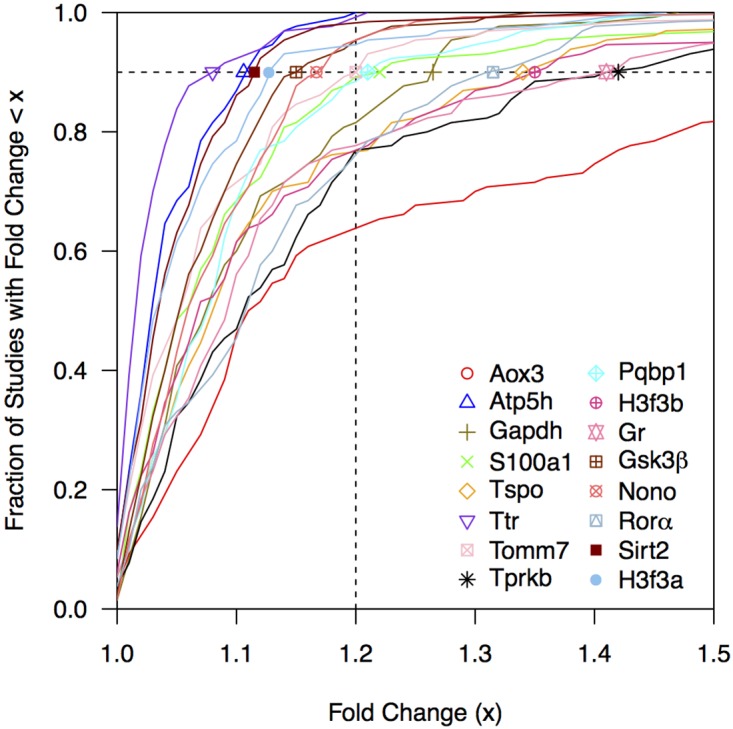
Fold-changes observed in 130 mouse liver transcriptome experiments. For each gene, we calculated and plotted the cumulative distribution of the fold-changes observed in a large set of 130 transcriptome experiments using mouse liver tissue. We considered candidate genes to pass this assessment if their fold-change was < 1.2 in 90% of all studies (indicated by dashed lines).

To assess the stability in gene expression of our candidate genes on an even larger scale, we used the data mining software Genevestigator to retrieve fold-changes for each candidate gene in a large series of 130 RNA-sequencing and DNA microarray experiments using mouse liver tissue. The cumulative distributions of these fold-changes are shown in Fig 4. In line with our previous results, most genes were found to have rather small fold-changes in the majority of studies. The gene with the least fold-change overall was *Ttr*, followed by *Atp5h* and *H3f3a*. For all of those genes we found that the fold-change was below 1.15 in more than 90% of all studies. In contrast, the fold-change reported for the gene *Aox3* was above 1.15 in more than 50% of the cases. We considered all genes to pass this assessment if their fold-change was quantified below 1.2 in more then 90% of all studies. Two genes, *Atp5h* and *Ttr* actually showed a fold-change of maximal 1.2 over all experiments (Fig 4).

### Selection of a final set of normalisation genes

A summary of our results is given in Table 1. Overall, only three genes (*Atp5h*, *Gsk3β*, and *Sirt2*) passed all of our assessments indicating that their expression was not affected by a wide variety of conditions including circadian changes and age. We thus consider the set of these three genes as our core normalisation set. An additional four genes (*Nono*, *Tprkb*, *Tspo*, and *Ttr*) passed all but one assessment, and we will consider them, together with the core genes, as an extended set of normalisation genes. Importantly, our set of normalisation genes does not include *Gapdh*, since the expression of this gene was found to be rather variable in several cases (Table 1). This thus suggests that additional power to detect true changes in expression can be gained by using the normalisation genes recommended here, instead of *Gapdh* alone.

### Identification of age-related changes between 3 and 24 month-old mice

We next analysed all of the other genes from our Nanostring experiment for age-related changes in their expression. To do so, we normalised the raw data based on the geometric mean of the normalisation constants obtained individually for each of the seven genes in our extended set [26] [36]. To obtain the most pronounced differences, we decided to compare 3 with 24 month-old mice. The analysis confirmed that most (32) of the 36 genes analysed in our experiment were not or only faintly affected by age at a Bonferroni-corrected p-value for multiple comparisons of *p* > 0.0014 using repeated-measures ANOVA (N = 24; S3 Fig). Hence, the statistical methodology was powerful enough to cope with the experimental noise observed in our experiment considering that many of the genes were expressed with circadian amplitude.

But this stringent way of normalisation also identified four genes for which expression was significantly affected by age at the p-value threshold of *p* < 0.0014 (Repeated-measures ANOVA, N = 24; S3 Fig) and 1.25 to 1.52 fold changes (Table 2). By contrast, there were no such significant changes comparing 3 with 12 month-old mice (S4 Fig), suggesting that the identified changes in the four genes were emerging at a later stage and hence represent age-related changes in expression. Genes found to increase their expression in 24 month-old mice were the *Casein kinases 1δ* and *ϵ*, and the DBHS family factor *Sfpq*, while the glucose transporter *Glut2* was decreased in its expression.

**Table 2:** Significant age-related changes 3 versus 24 month-old mice.

Gene	p-Value (r-m ANOVA)	Fold Change (Nanostring)	Fold Change (RT-PCR)
*Csnk1ϵ*	0.00018	1.52	1.77
*Glut2*	0.00035	0.74	0.80
*Sfpq*	0.0005	1.33	1.38
*Csnk1δ*	0.0012	1.25	1.42

p-Value: p-value as obtained by the repeated-measures ANOVA; Fold Change: average fold-change as measured by the Nanostring hybridisation experiment, or real-time PCR.

### Verification of age-related changes by real-time PCR and Western blot

**Fig 5:**
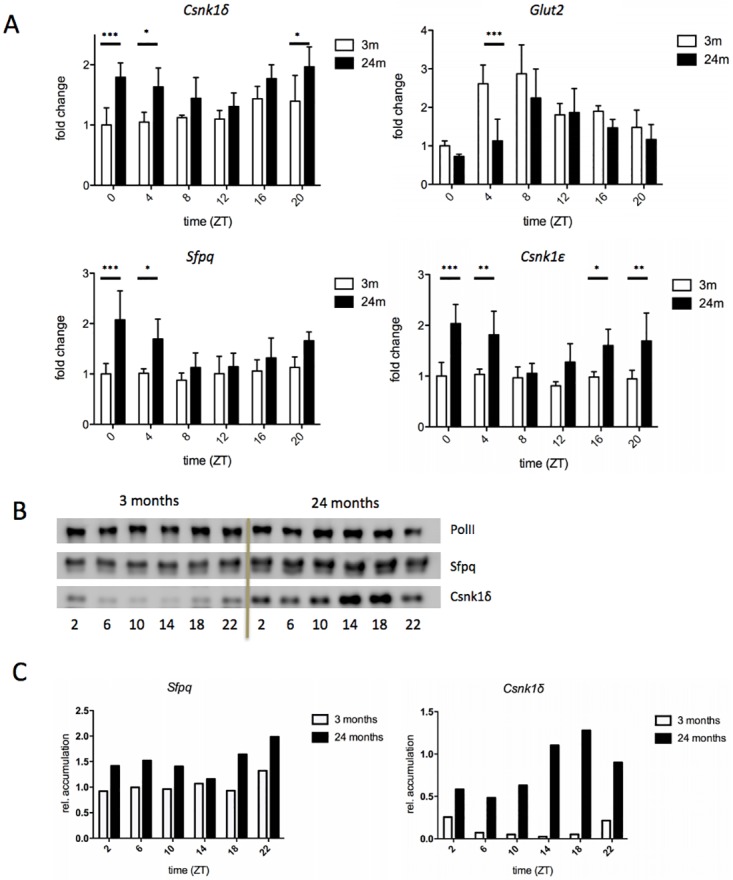
Validation of gene expression by real-time PCR and protein accumulation by Western blot. A) fold-change of expression of the indicated genes over the circadian cycle as measured by real-time PCR; B) accumulation of Csnk1*δ* and Sfpq in nuclear extracts with the controls RNA polymerase II (RPII); C) quantification of Sfpq and Csnk1*δ* against RPII. Mean ± STD, n = 4, Bonferroni Post test, *: p < 0.05, **: p < 0.01, ***: p < 0.001.

To independently verify our findings, we next quantified the expression of the age-affected genes by real-time PCR (Fig 5A, Table 2). Surprisingly the three genes identified as up-regulated in 24 month-old mice displayed a similar pattern of accumulation over the circadian cycle, i.e. a more or less constant accumulation in 3 month-old animals but rhythmic accumulation with similar phase in 24 month-old animals. By contrast, the circadian amplitude of *Glut2* was reduced with advancing age. We found a strong interaction between the expression series of all genes and age (two-way ANOVA, *p* < 0.001, Fig 5A). Not surprisingly, time was also identified as a significant source of variation (two-way ANOVA, *p* < 0.01).

To verify that the observed changes in expression translate into real differences in protein abundance, we conducted Western blot experiments using nuclear extracts obtained from livers of 3 and 24 month-old mice. A protein chosen as control, RNA Polymerase II (RPII), did not display gross changes in its accumulation over time and age and was consequently exploited for normalization purposes (Fig 5B). Using this method, an increase of Csnk1*δ* and Sfpq in the nucleus became apparent with fold-change of 1.47 for Sfpq and 7.44 for Csnk1*δ* averaged over all 6 time points (Fig 5C). These results confirm that the subtle increases as observed for gene expression result in detectable increases of the corresponding protein levels, and thus demonstrate the biological relevance of these changes.

## Discussion

Global and more restrained transcriptome analyses are now routinely used and new methods such as RNA-sequencing and Nanostring hybridization, respectively, are very sensitive to detect changes of a given transcriptome in response to different experimental conditions. However, accurate inference always relies on proper normalisation, without which the inferred expression changes may only be due to experimental noise [19]. Here, we report a set of normalisation genes our analysis suggests is adequate to study gene expression in the mouse liver [37] [38]. These genes were selected using a rigorous statistical approach, the centered log-ratio analysis, which is commonly used when dealing with compositional data, and which is a natural extension of the often used fold-change analysis to more than two experimental settings [20]. Applying this statistical framework, we tested a set of candidate genes for low variance in expression against a variety of conditions.

First, and using data we generated from both the RNA-sequencing and Nanostring hybridization experiments, we showed that the expression of the proposed set of normalisation genes is sufficiently large for accurate quantification, yet stable throughout the day (i.e. to be unaffected by the circadian clock) and does not change with age. Second, and using previously published data sets, we could not only confirm these results, but also further generalize the usefulness of our set of genes by showing that their expression was also constant across the sexes and across different mouse tissues. Finally, we showed that these genes were constant in a majority of conducted transcriptome experiments with mouse liver tissue to date. Hence, this set of normalisation genes represents the way to cope with the problem of transcriptional noise as observed with increased age [16].

Our final set of seven normalisation genes consisted of the genes *Atp5h*, *Gsk3β*, *Nono*, *Sirt2*, *Tprkb*, *Tspo*, and *Ttr*. Interestingly, we found the gene *Gapdh*, which has been commonly used as the single gene for normalisation [17], to be much more variable than these genes, including when comparing expression patterns between mice of different age. Hence, we can confirm previous findings that *Gapdh* may not be an ideal gene for normalisation in this tissue [18]. In addition, and as was previously reported, we found that due to experimental noise, a single gene is unlikely to be sufficient for proper normalisation. Instead, and following previous recommendations [39], we advocate using the geometric mean of a number of marker genes, ideally the seven genes as identified in our study. In this way, the impact of one or two outliers of expression is mathematically filtered out, reducing the noise of the analysis.

In order to identify differences in gene expression between 3 and 24 month-old mice, we thus first calculated the normalisation constants for each marker gene individually, and then used the geometric mean of those constants for normalisation [39]. Employing here for the first time such a stringent normalisation method on circadian expression data, we discovered the expression of a new class of genes to be affected by age, namely *Csnk1δ* and *Csnk1ϵ* (Fig 5A). *Csnk1δ* and *Csnk1ϵ* phosphorylate components of the circadian oscillator to set the pace of oscillation [10] [11]. Interestingly, a mutation in *Csnk1ϵ* reducing its enzymatic activity was identified in hamster [40]. This mutated form of Csnk1*ϵ* was later found to increase the life expectancy of the hamster [41]. Hence, we may speculate that an age-related increase of Csnk1 activity could be harmful for an organism over time. However, further experiments are necessary to understand the impact of this increase in expression of *Csnk1δ* and *Csnk1ϵ* on the post-translational regulation of the circadian oscillator with advancing age. Also, the effect of this increase on other described targets of Csnk1 phosphorylation such as p53 and *β*-catenin has to be considered [42] [43]. By contrast, an influence of *Sfpq* or other DBHS family factors on the aging process has not yet been observed [12] and it is worth to further investigate the role of these factors in 24 month-old and even older mice.

Surprisingly, the expression of *Glut2* was decreased (Fig 5A). The expression of *Glut2* was previously shown to be regulated by BMAL1 affecting the glucose metabolism of the liver [44]. Hence, it is tempting to speculate that the reduction of *Glut2* was mediated by a reduction of the activity of the BMAL1/CLOCK heterodimer. However, we did not detect down-regulation of these factors on the level of mRNA accumulation (S3 Fig). Also, we did not observe down-regulation of any other BMAL1/CLOCK-regulated gene such as *Per1*, *Per2*, *Per3*, *Gm129*, *Dbp*, or *Tef* (S3 Fig), rendering it likely that BMAL1/CLOCK activity did not decline in 24 month-old mice. Taken together, the reason for the down-regulation of *Glut2* remains unknown. On the other hand, the three genes significantly up-regulated with age showed a circadian accumulation profile in 24 month-old mice reminiscent of genes regulated by the stabilizing loop [7] [8] [9] (Fig 5A). Again, we did not observe up-regulation of genes involved in this kind of regulation such as *Rorα*, *Rorγ*, *Pparα*, or *Pparγ*, or down-regulation of the corresponding repressors *Rev-Erbα* and *Rev-Erbβ* (S3 Fig). Interestingly, in a previous study it was found that Ppar*α* and Ppar*γ* occupied more chromatin binding sites in the livers of 21 month-old mice affecting the lipid metabolism and causing steatosis [27]. This observation could point towards post-translational processes influencing the activity of the aforementioned nuclear receptors. However, further analysis is necessary to understand the impact of the identified age-related changes on the circadian oscillator in other organs and the overall organism.

## Conclusion

Here we report a set of seven genes suitable for normalisation of transcriptome analysis experiments using mouse liver. These genes (*Atp5h*, *Gsk3β*, *Nono*, *Sirt2*, *Tprkb*, *Tspo*, and *Ttr*) were selected based upon a rigorous statistical evaluation of their overall expression levels and their variance in expression across a multitude of experimental conditions, including circadian changes, sex and, most importantly, age. We found these genes to be more appropriate for normalisation than using the gene *Gapdh*, which exhibited more variance in its expression. Our results suggest that the sensitivity of assays to study differences in gene expression in mouse liver can be much improved by normalising the data with the larger set of marker genes proposed here. With the increased robustness based on our normalisation, we were able to detect age-related changes of the circadian regulatory network.

## Supporting Information

S1 Fig Comparison of gene expression changes between different age classes. The data were retrieved from the RNA-sequencing experiment. Shown are the fold-changes in gene expression compared between all age groups for the candidate genes. All fold-changes were calculated after clr normalisation.
(TIF)

S2 Fig Correlation analysis of the expression of candidate genes. Shown are the pair-wise correlations between all pairs of candidate genes. All correlations were calculated from the raw counts from the Nanostring hybridization experiment after normalisation with the positive controls, but without any further normalisation. A colour-coded scale represents maximal correlation (positive blue, negative red) to no correlation (white).
(TIF)

S3 Fig Comparison of 3 and 24 month-old mice. Compared are the normalised mRNA counts from the Nanostring hybridization experiment with 12 different time points in duplicates. The mRNA counts at the same time points are connected by a line. A red line indicates the difference in the mean expression between the two different age classes. Significance was assessed by repeated-measures ANOVA and the p values indicated. Significant p-values (*p* < 0.0014) are indicated in red.
(TIF)

S4 Fig Comparison of 3 and 12 month-old mice. The normalised mRNA counts from the Nanostring hybridization experiment of the 36 genes were compared from 3 and 12 month-old animals. The mRNA counts at the same time points are connected by a line. A red line indicates the difference in the mean expression between the two different age classes. Significance was assessed using repeated-measures ANOVA and the p values indicated.
(TIF)

S1 Table List of identified genes in the RNA-sequencing experiment comparing 3 and 15 month-old animals. (XLS)

S2 Table List of Nanostring probe pairs and their location within the genes. (XLS)

S3 Table List of real-time PCR primer and probes. (PDF)
