# Supplementary figures and images for: Normalisation against Circadian and Age-Related Disturbances Enables Robust Detection of Gene Expression Changes in Liver of Aged Mice
Source: PLoS One. 2017 Jan 9;12(1):e0169615. doi: 10.1371/journal.pone.0169615 (PMC5222604; doi:10.1371/journal.pone.0169615)

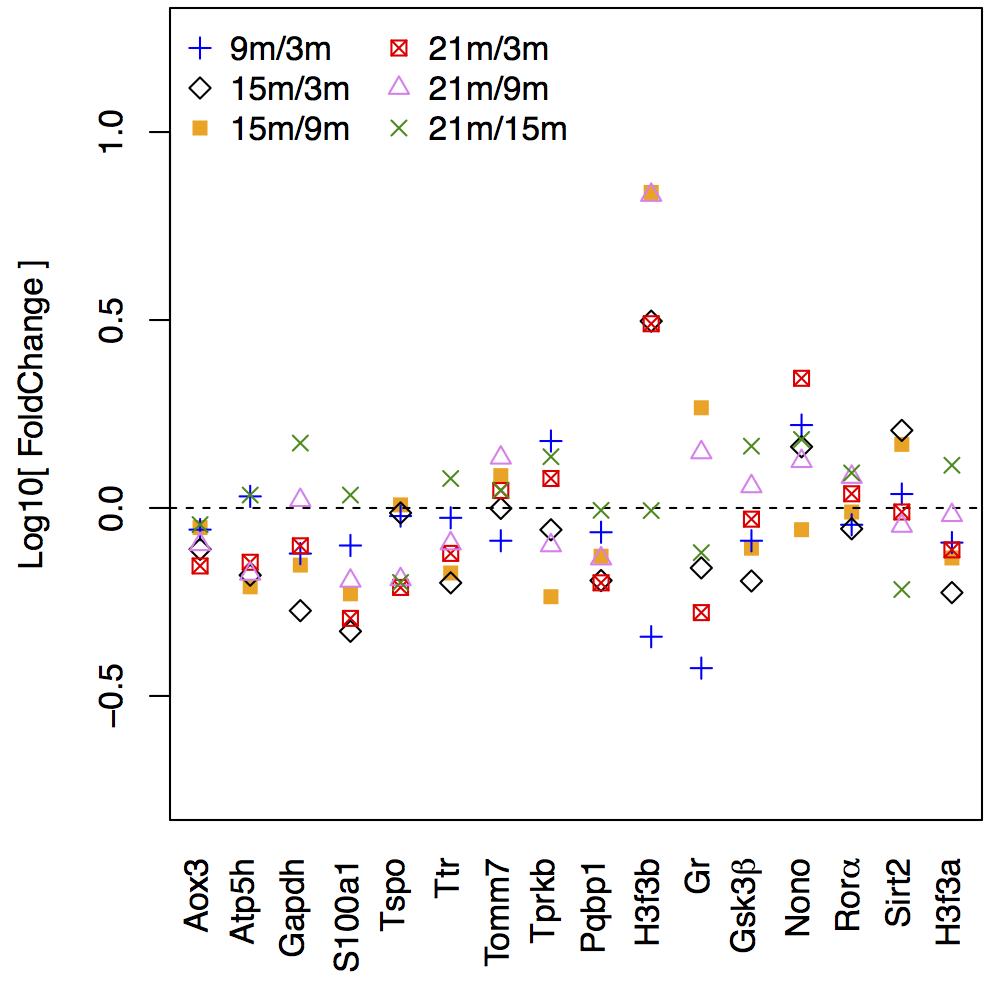

Supplement: S1 Fig — The data were retrieved from the RNA-sequencing experiment. Shown are the fold-changes in gene expression compared between all age groups for the candidate genes. All fold-changes were calculated after clr normalisation. (TIF) [file pone.0169615.s001.tif]

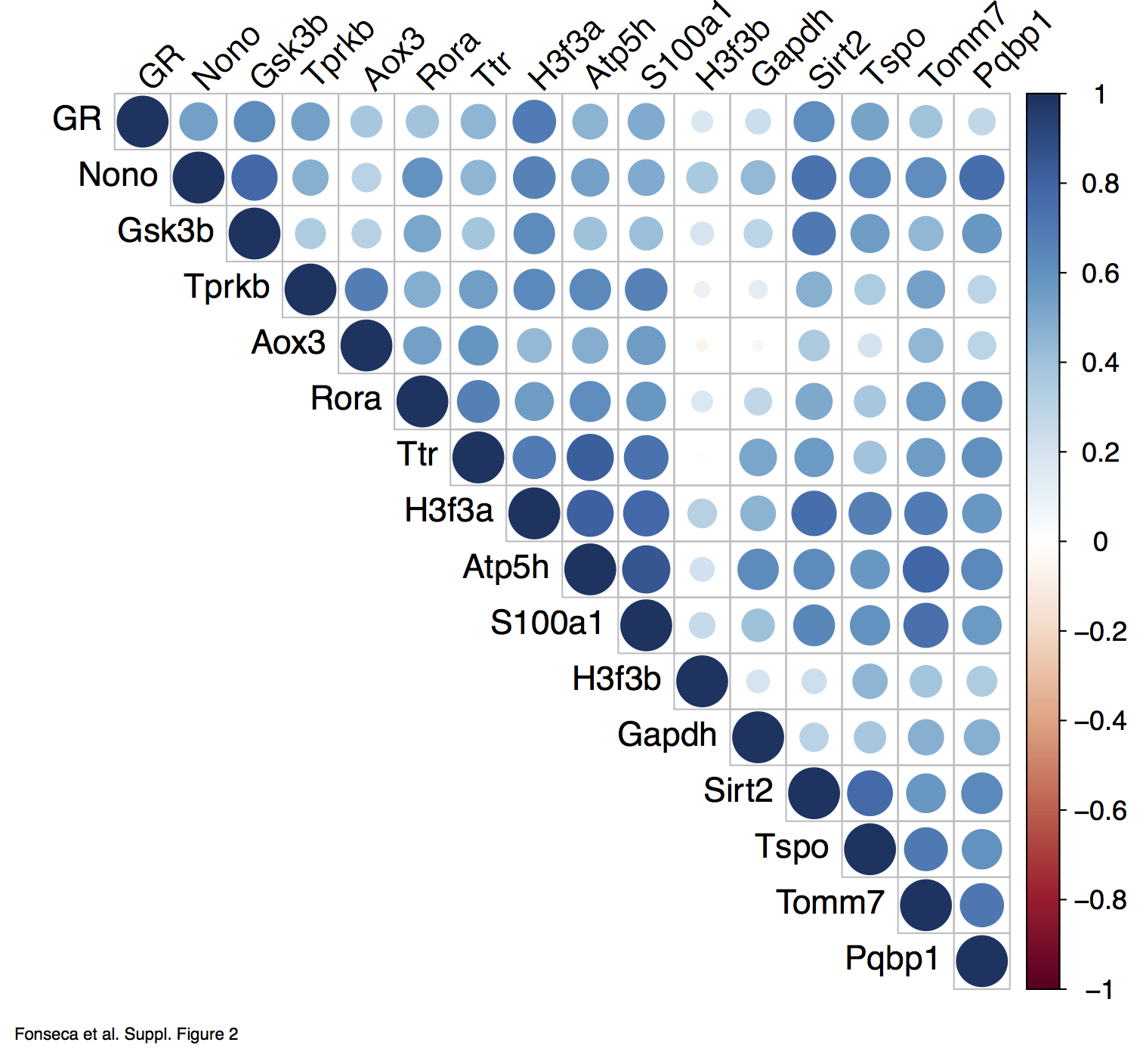

Supplement: S2 Fig — Shown are the pair-wise correlations between all pairs of candidate genes. All correlations were calculated from the raw counts from the Nanostring hybridization experiment after normalisation with the positive controls, but without any further normalisation. A colour-coded scale represents maximal correlation (positive blue, negative red) to no correlation (white). (TIF) [file pone.0169615.s002.tif]

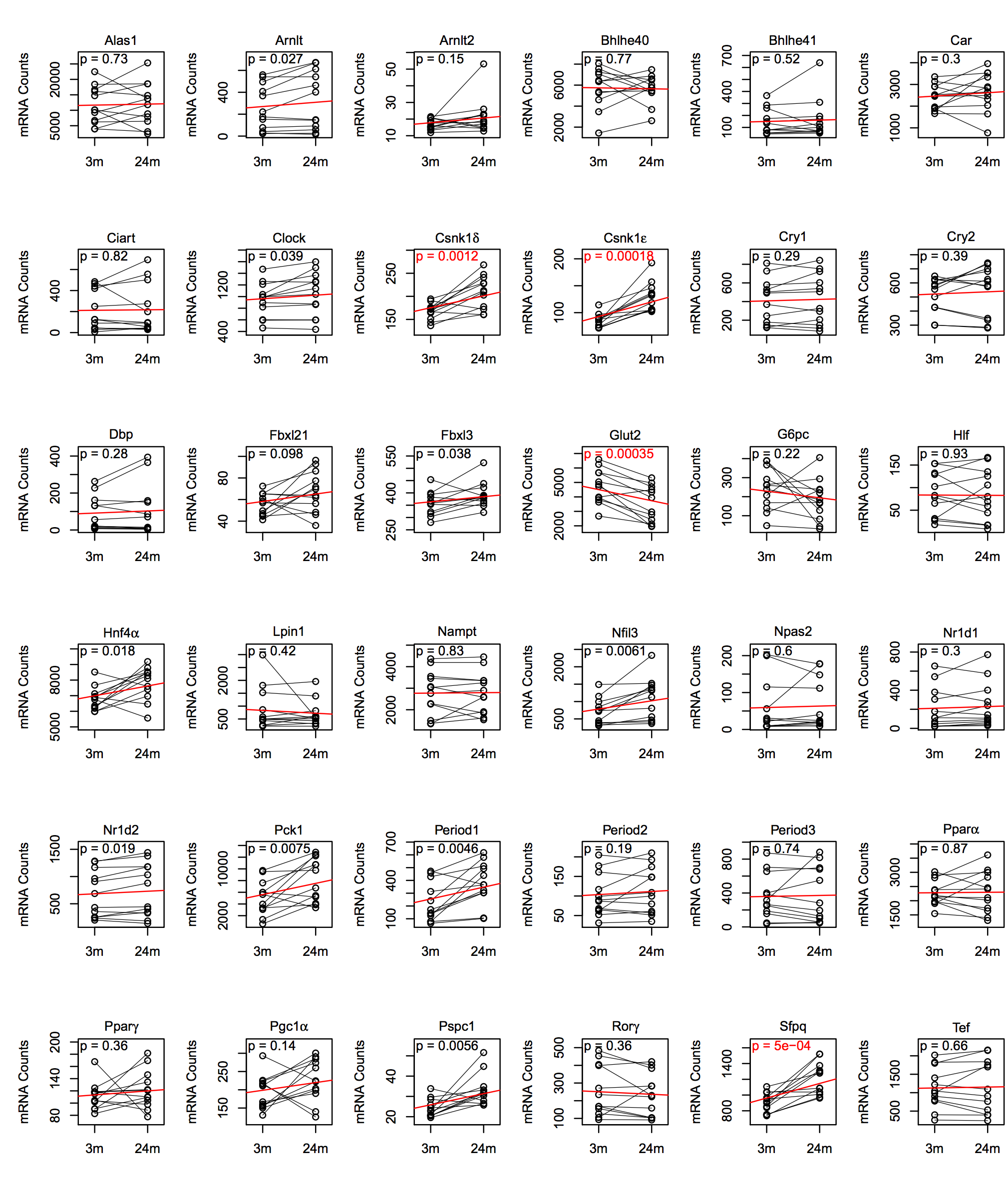

Supplement: S3 Fig — Compared are the normalised mRNA counts from the Nanostring hybridization experiment with 12 different time points in duplicates. The mRNA counts at the same time points are connected by a line. A red line indicates the difference in the mean expression between the two different age classes. Significance was assessed by repeated-measures ANOVA and the p values indicated. Significant p-values (p < 0.0014) are indicated in red. (TIF) [file pone.0169615.s003.tif]

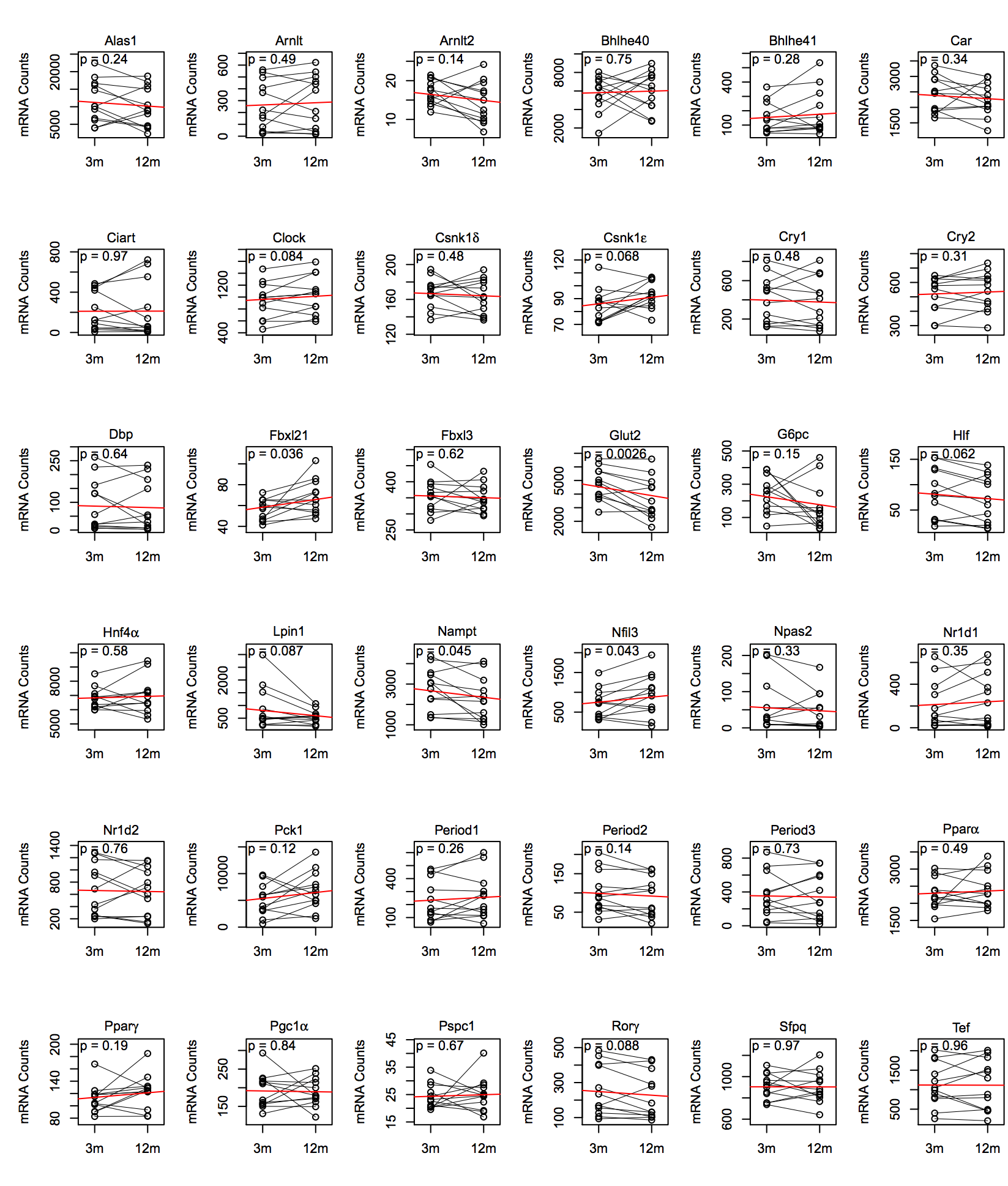

Supplement: S4 Fig — The normalised mRNA counts from the Nanostring hybridization experiment of the 36 genes were compared from 3 and 12 month-old animals. The mRNA counts at the same time points are connected by a line. A red line indicates the difference in the mean expression between the two different age classes. Significance was assessed using repeated-measures ANOVA and the p values indicated. (TIF) [file pone.0169615.s004.tif]
