# Supplementary material for: Normalisation against Circadian and Age-Related Disturbances Enables Robust Detection of Gene Expression Changes in Liver of Aged Mice
Source: PLoS One. 2017 Jan 9;12(1):e0169615. doi: 10.1371/journal.pone.0169615 (PMC5222604; doi:10.1371/journal.pone.0169615)
Supplement: S3 Table — (PDF) [file pone.0169615.s007.pdf]

Real-time PCR probes for mRNA determination

FAM: 6-fluorescin; YY: Yakima yellow; BHQ1: Black hole quencher type 1

Genes for normalization:

Atp5h
FW: TGC CCT GAA GAT TCC TGT GCC T
RV: ACT CAG CAC AGC TCT TCA CAT CCT
TM: YY-TCT CCT CCT GGT CCA CCA GGG CTG TGT-BHQ1

Gsk3β
FW: CCA CCT CCT TTG CGG AGA GC
RV: CTG TGG TTA CCT TGC TGC CAT CT
TM: YY-TGC AAG CCA GTG CAG CAG CCT TCA GCT-BHQ1

SirT2
FW: CAG GCC AGA CGG ACC CCT TC
RV: AGG CCA CGT CCC TGT AAG CC
TM: FAM-TGA TGG GCC TGG GAG GTG GCA TGG A-BHQ1

Circadian genes:

Csnk1δ
FW: GGA ACG AGA ACG GAA AGT GAG TAT GC
RV: TGT CGG CCC GTG AGA TCA GA
TM: FAM-CAC CGT GGG GCC CCA GTC AAC GT-BHQ1

Csnk1ε
FW: CGC CAA TCG AGG TCC TCT GCA A
RV: CGG AAG AGC TGG CGC AGG TA
TM: FAM-ACT TCT GCC GCT CCC TGC GGT TCG A-BHQ1

Glut2
FW: TTC CTT CCA GTT CGG CTA TG
RV: GAA CAC CCA AAA CAT GTC GAT
TM: FAM-CAT CGG TGT GAT CAA TGC ACC TCA-BHQ1

Sfpq
FW: ATA CGG AGA ACC CGG AGA AG
RV: CTC AGC TTT GGC GAT TTC AG
TM: FAM-TGA ACC CGA ACC CTT TGC CTT TG-BHQ1
